# The changes in health service utilisation in Malawi during the COVID-19 pandemic

**DOI:** 10.1371/journal.pone.0290823

**Published:** 2024-01-17

**Authors:** Bingling She, Tara D. Mangal, Anna Y. Adjabeng, Tim Colbourn, Joseph H. Collins, Eva Janoušková, Ines Li Lin, Emmanuel Mnjowe, Sakshi Mohan, Margherita Molaro, Andrew N. Phillips, Paul Revill, Robert Manning Smith, Pakwanja D. Twea, Dominic Nkhoma, Gerald Manthalu, Timothy B. Hallett

**Affiliations:** 1 Department of Infectious Disease Epidemiology, Imperial College London, London, United Kingdom; 2 Institute for Global Health, University College London, London, United Kingdom; 3 College of Medicine, University of Malawi, Lilongwe, Malawi; 4 Centre for Health Economics, University of York, York, United Kingdom; 5 Centre for Advanced Spatial Analysis (CASA), University College London, London, United Kingdom; 6 Department of Planning and Policy Development, Ministry of Health and Population, Lilongwe, Malawi; The Uniersity of British Columbia, ETHIOPIA

## Abstract

**Introduction:**

The COVID-19 pandemic and the restriction policies implemented by the Government of Malawi may have disrupted routine health service utilisation. We aimed to find evidence for such disruptions and quantify any changes by service type and level of health care.

**Methods:**

We extracted nationwide routine health service usage data for 2015–2021 from the electronic health information management systems in Malawi. Two datasets were prepared: unadjusted and adjusted; for the latter, unreported monthly data entries for a facility were filled in through systematic rules based on reported mean values of that facility or facility type and considering both reporting rates and comparability with published data. Using statistical descriptive methods, we first described the patterns of service utilisation in pre-pandemic years (2015–2019). We then tested for evidence of departures from this routine pattern, i.e., service volume delivered being below recent average by more than two standard deviations was viewed as a substantial reduction, and calculated the cumulative net differences of service volume during the pandemic period (2020–2021), in aggregate and within each specific facility.

**Results:**

Evidence of disruptions were found: from April 2020 to December 2021, services delivered of several types were reduced across primary and secondary levels of care–including inpatient care (-20.03% less total interactions in that period compared to the recent average), immunisation (-17.61%), malnutrition treatment (-34.5%), accidents and emergency services (-16.03%), HIV (human immunodeficiency viruses) tests (-27.34%), antiretroviral therapy (ART) initiations for adults (-33.52%), and ART treatment for paediatrics (-41.32%). Reductions of service volume were greatest in the first wave of the pandemic during April-August 2020, and whereas some service types rebounded quickly (e.g., outpatient visits from -17.7% to +3.23%), many others persisted at lower level through 2021 (e.g., under-five malnutrition treatment from -15.24% to -42.23%). The total reduced service volume between April 2020 and December 2021 was 8 066 956 (-10.23%), equating to 444 units per 1000 persons.

**Conclusion:**

We have found substantial evidence for reductions in health service delivered in Malawi during the COVID-19 pandemic which may have potential health consequences, the effect of which should inform how decisions are taken in the future to maximise the resilience of healthcare system during similar events.

## Introduction

During the early phases of the COVID-19 pandemic, there were concerns that there would be large disruptions to healthcare, especially in settings with healthcare systems that are already stretched. These could arise from disruptions to health service delivery due to reduced clinic times or health workforce being deployed to COVID-19 response activities, or from persons becoming less willing or able to seek care due to travel restrictions and fears of being infected with the COVID-19 [1–6].

Retrospective evaluations have since found that disruptions to healthcare services did occur, especially in low-income and middle-income countries (LMICs) in Africa, South-East Asia and Eastern Mediterranean Regions [1–3, 7–15], including Malawi [4–6, 16–19], which is a low-income country in sub-Saharan Africa with a fragile healthcare system [20]. These studies have described disruptions to services relating to HIV, TB, routine immunisation, maternal and child health care, and outpatient visits (e.g. 39% decrease in HIV tests and 19.1% decrease in TB treatment registration by February 2021 in 8 facilities in Lilongwe, Malawi [17]). Notably, the published studies for Malawi have mostly focused on small subsets of facilities (e.g., a central hospital, selected facilities in one district) or small subsets of service types, meaning that a full picture of the changes experienced across the healthcare sector is in need for better understanding the COVID-19 disruptions.

**Fig 1:**
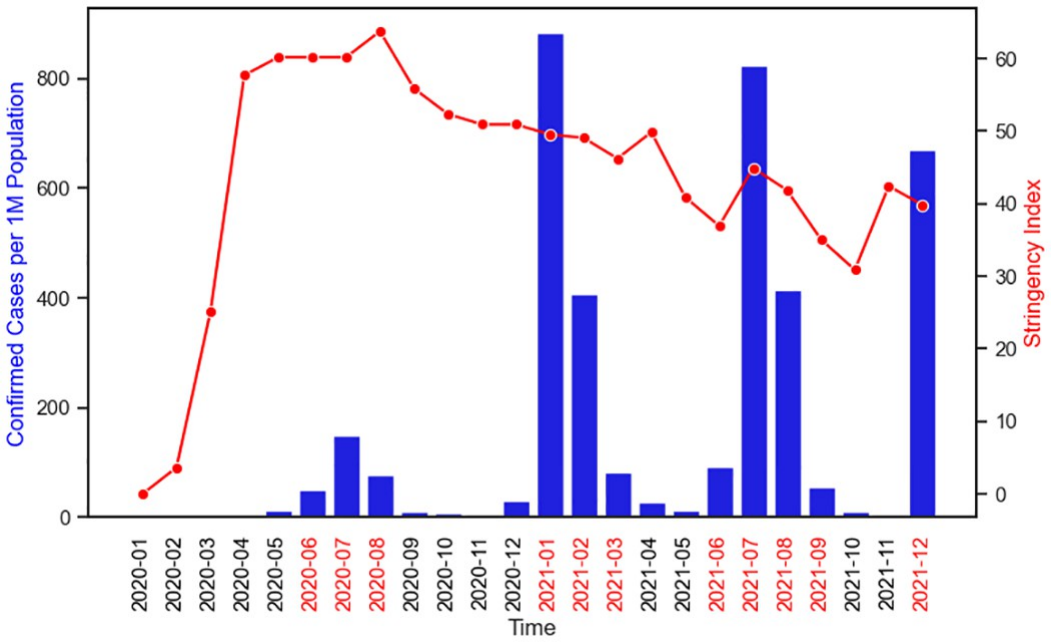
COVID-19 confirmed cases and stringency index in Malawi, 2020–2021. There have been four waves (> 49 cases per 1 million persons) as indicated in red time labels. The data source is [22].

The electronic health information management systems for all healthcare-related services in Malawi presents an opportunity to present such an analysis. From 2 April 2020, when Malawi registered the first confirmed case [21], to December 2021, Malawi has experienced four waves of the COVID-19 pandemic (Fig 1) with concomitant periods of government-mandated restrictions in multiple activities. The government response included school closure, non-essential workplaces closing, cancelling public events, restrictions on gathering and public transportation, and international travel control, the overall level of which is measured by an aggregated Stringency Index measure ranging from 0 to 100 [21, 22]; April-September 2020 had the strictest restrictions with Stringency Index over 55.

In this study, we draw on the routinely collected records in Malawi to: (i) describe routine usage at each facility level (i.e., the level of health care) within the healthcare system in the pre-pandemic period of 2015–2019; (ii) test for evidence of changes in the pandemic period of 2020–2021; (iii) quantify the total deficits in health service utilisation in 2020–2021.

## Materials and methods

Health services in Malawi are provided by public (free-of-charge), private for profit and private-not-for-profit sectors, and health care are delivered by 1548 health facilities through a three-tier system namely: primary, secondary and tertiary, where lower levels provide referral services to higher levels [23–25].

### Data

From February to May 2022, we extracted 2015–2021 health service utilisation data from reports provided by Malawi DHIS2 (District Health Information Software) system [26] and the HIV & AIDS and Viral Hepatitis Department. These reports record the numbers (or frequencies) of health services delivered at each health facility in each month (or year). Table 1 lists the 20 service types included in this research, along with their descriptions and data sources.

**Table 1:** Service types, data sources, reports and reporting rates, and data elements.

Service type*	Data source	Report	Reporting rate†	Data element/Service description
InpatientDays	DHIS2	HMIS	94.22%	Total Inpatient Days
IPAdmission	DHIS2	HMIS	94.22%	Total number of Admissions (including Maternity) and Discharges
OPD	DHIS2	HMIS	94.22%	Total number of Outpatient Attendance
U5Malnutr	DHIS2	HMIS	94.22%	Total number of Malnutrition—New Case (under 5)
Delivery	DHIS2	Maternity	95.97%	Total cases of Delivery Mode Spontaneous Vagina, Delivery Mode Breech, Delivery Mode Vacuum Extraction
Csection	DHIS2	Maternity	95.97%	Total cases of Delivery Mode Caesarian Section
FamPlan	DHIS2	Family Planning	79.31%	Contraception cases of all methods listed in the report, Numbers of New and Old Clients Counseled re. family planning
AntenatalTotal	DHIS2	HMIS	94.22%	Total Antenatal Visits
EPI	DHIS2	EPI (New)	84.39%	Cases of Children vaccinated with BCG, Children vaccinated with DPT-HepB-Hib, Children vaccinated with MR, Measles Childhood Vaccination, OPV0/1/2/3 Childhood Vaccination, PCV1/2/3 Childhood Vaccination, Children vaccinated with ROTA, TT Vaccination for Non Pregnant Women/Pregnant Women, Total Td for non-pregnant/pregnant women
STI	HIV Dept	STI	N/A	Cases of sexually transmitted infections treatment for Age group A (0–19 years), Age group B (20–24 years) and Age group C (25+ years)
AccidentsandEmerg	DHIS2	HMIS	94.22%	Numbers of Patients re. Road Traffic Accidents and Common Injuries and Wounds (except RTA)
TBNew**	DHIS2	TB Case Findings (New)	84.10%	TB New Case findings of all categories in the report
VCTTests	HIV Dept	HTS	N/A	Total numbers of HIV tests re. Single test negative, Test 1&2 negative, Single test positive, Test 1&2 positive, Test 1&2 discordant
MaleCirc	DHIS2	VMMC	54.69%	Total numbers of male circumcisions re. Location total, Total With 1st Visits, Total With 2nd Visits in the report
NewAdult	HIV Dept	ART Visits	N/A	Number of antiretroviral therapy (ART) initiation for adults (15+ yrs)
EstAdult	HIV Dept	ART Visits	N/A	Number of ART follow-up visits for adults (15+ yrs)
PMTCT	HIV Dept	ART Visits	N/A	Number of ART initiation/follow-up visit for pregnant_female
Peds	HIV Dept	ART Visits	N/A	Number of ART initiation for pediatrics (0–14 yrs), Number of ART follow-up visits for pediatrics (0–14 yrs)
DentalAll	DHIS2	Dental	22.11%	Total number of patients seen
MentalAll	DHIS2	Mental	49.65%	Total number of patients seen by Mental Health Clinical Officer and by Mental Health Nurse, Cases of all diagnosis categories in the report

*The selection of **service types** follows that used by Berman et al [28], which was based on Malawi’s essential health package [24], and has been adapted considering data sources and availability. The **data elements** describe the measures of utilisation for each service type.

**We extracted monthly data for all service types except TBNew, for which yearly data were provided instead.

^**†**^The **reporting rate** is the monthly average reporting rate between 2015 and 2019 over primary and secondary levels (1a, 1b, 2); for TB, it is yearly average between 2017 and 2019.

There are 955 health facilities currently registered in DHIS2 system, consisting of 855 health centres, maternity facilities, clinics and dispensaries grouped in this analysis as facility level 1a, 69 community/rural hospitals and CHAM (Christian Health Association of Malawi) hospitals as facility level 1b and 24 district hospitals as facility level 2. Other facilities include 2 health posts, 4 central hospitals and Zomba Mental hospital, which have limited data in DHIS2 and are therefore excluded from the main analysis (These hospitals use an older version of DHIS that is not linked to DHIS2). Within Malawi healthcare system, levels 1a and 1b are the primary levels of health care delivery and level 2 is the secondary level of health care delivery [23, 24].

The reports in DHIS2 come with a reporting rate indicator signalling that the record for a facility (that is expected to report) in a particular month is reported or not [26, 27], which we use to infer Type A missing data if it is not reported. Table 1 presents the average monthly reporting rate per available report in the pre-pandemic period of 2015–2019. These less than 100% reporting rates indicate Type A missing data.

**Fig 2:**
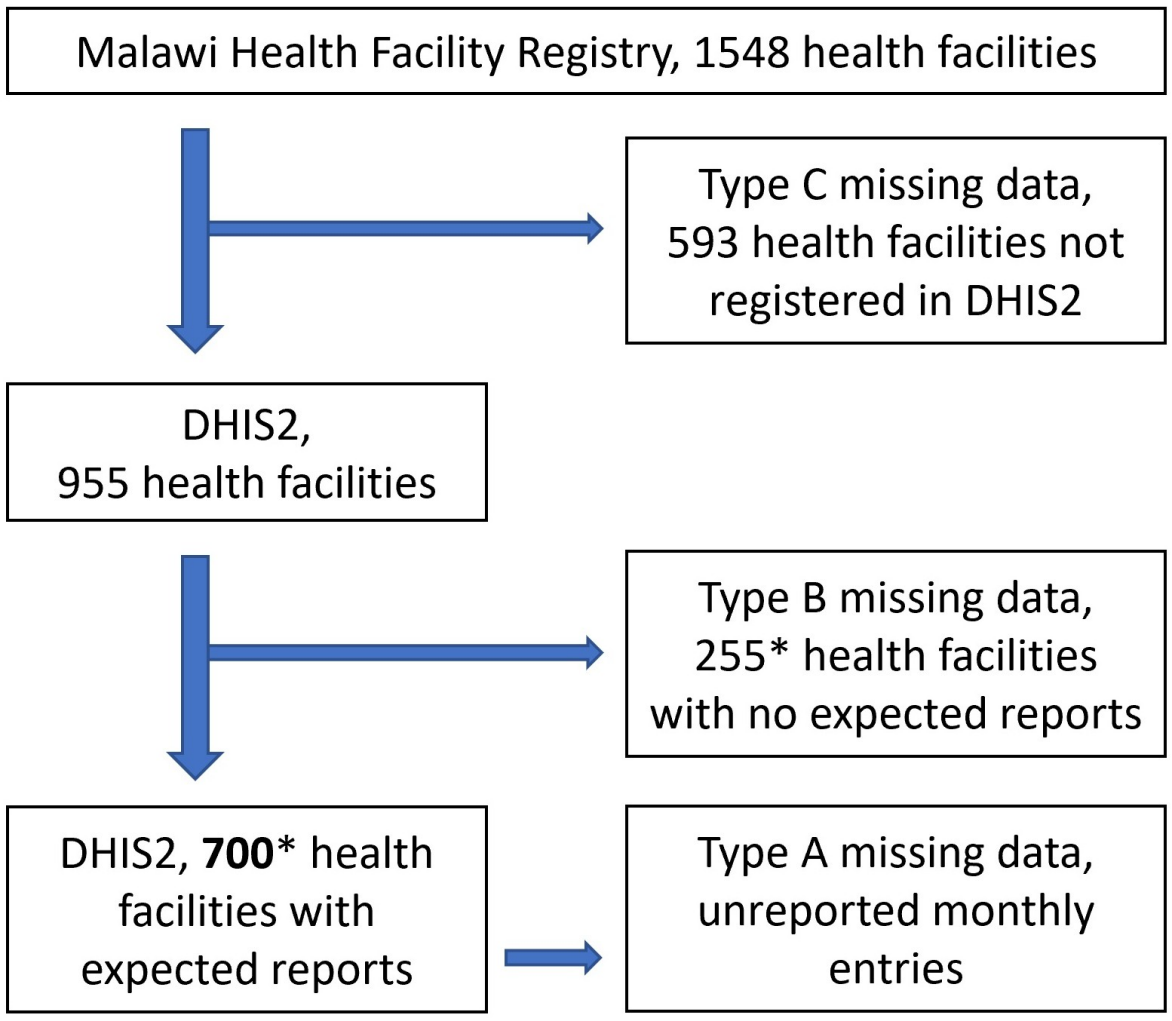
Three types of missing data for health service records in Malawi DHIS2 system. *These are approximate numbers, as the numbers of facilities with expected reports vary among different reports (e.g., HMIS report: 683 facilities, Maternity report: 575, EPI report: 694).

Furthermore, we identified Type B missing data due to facilities in DHIS2 having no records at all as they have no expected reports [27], and Type C missing data due to facilities that do exist but are not yet registered in DHIS2 thus having no records at all either [25]. All three types of missing data could lead to underestimation of total service usage, and the literature mainly considers Type A [11]. Fig 2 illustrates the facility counts and three types of missing data. We included the 700 (approximately) health facilities with expected reports in this research, which have Type A missing data.

To describe the change of service utilisation during the COVID-19 pandemic, we divided the data into two parts: the pre-pandemic period (years 2015–2019, inclusive) and the pandemic period (years 2020 & 2021).

### Data preparation

First, records with a z-score greater than 3.0 [29] were treated as extreme outliers, which accounted for 2.18% of all reported data, and were replaced with the mean of reported monthly values excluding outliers at that facility. (More specifically, to find extreme outliers, we firstly drew scatter plots and then did experiments on 2 z-score and 3 z-score methods for each service type and each facility type. We found that 3 z-score is helpful to identify extreme values as well as keeping reasonably large values, as recommended by WHO [29]. The proportions of outliers are 4.42% and 2.18% for the two methods, respectively.).

Then considering reporting completeness (Type A missing data) and its possible impact on the analysis and results, we prepared two datasets:

- The “Unadjusted” dataset, wherein reported counts were used without any further adjustment and unreported counts of services delivered in a facility in a particular month were treated as being equal to zero.
- The “Adjusted” dataset, wherein we aimed to: (i) fill-in unreported monthly values per facility, and (ii) carry forward the pattern of these adjustment into the pandemic period. Such adjustments were aimed for the facilities with expected reports (Fig 2) and for the services included in DHIS2 (Table 1). In respect of (i), we devised four adjustment methods M1-M4 that are based on reported values and apply them to different service types as appropriate. More specifically, M1 considers potential seasonal trends and makes interpolation from the same facility and same month from other years with reported data; M2 makes interpolation from the same facility from other months of the same year with reported data; M3 makes interpolation from the same facility from all other months and years with reported data; M4 makes interpolation from other facilities of the same type from all months and years with reported data. In respect of (ii), we then, under the assumption that the bias induced through the misreporting and its appropriate adjustment are the same in the period 2015–2019 as in 2020–2021, made adjustment for each service type at each facility level by applying method M5 that uses adjustment factors calculated from the adjustment results of pre-pandemic period. S1 File presents the formulas for M1-M5 methods and the implementation to each service type.

### Analysis

#### Describing health service utilisation in 2015–2019

Using the “Adjusted” datasets, we calculated monthly and annual frequencies for each service type at facility levels 1a, 1b and 2 to examine the service utilisation distribution at primary and secondary levels as well as evidence of temporal changes such as seasonal patterns.

#### Testing for evidence of reductions in services delivered during the COVID-19 pandemic

We defined three tests as follows.

Test One: Using the “Adjusted” dataset to examine the magnitude and duration of changes, we calculated the mean and standard deviation of frequency of each service type at each facility level in each calendar month over the years 2015–2019. For each monthly frequency of a service type at a facility level in 2020–2021, we considered there was substantial evidence for a reduction if it is below the mean by more than 2 standard deviations, small evidence for a reduction if it is below the mean by no more than 2 standard deviations, and no evidence for a reduction if it is not below the mean.

Test Two: Following the methods in Test One but using the “Unadjusted” dataset.

Test Three: Using the “Unadjusted” dataset, we restricted the data to those facilities maintaining 100% reporting, and then, for each service type, facility level and pandemic month, we calculated the per cent of facilities that reported reduced frequency (i.e., those have less frequency than the mean of the same months in pre-pandemic period). We considered there was substantial evidence for a reduction (of a service type at a facility level in a month) if the per cent is above 75%, small evidence for a reduction if the per cent is between 50% and 75%, and no evidence for a reduction if the per cent is below 50%.

We considered Test One to be the main signal of a reduction in service utilisation and used Test Two and Test Three to verify that results did not arise artefactually due to biases introduced in the process in the adjustments of the datasets.

#### Quantification in deficits of service utilisation during the COVID-19 pandemic

Using both “Adjusted” and “Unadjusted” datasets, we calculated the total cumulative net difference frequency for each service type in 2020–2021, compared to the averages of same months in 2015–2019.

## Results

### Health service utilisation before the COVID-19 pandemic, 2015–2019

**Fig 3:**
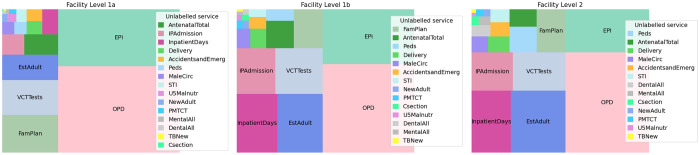
Average annual health service utilisation by service type and facility level 2015–2019. The frequency is calculated as the number of services delivered per 10,000 persons in Malawi, using “Adjusted” dataset. The area of each rectangle is proportional to the frequency of each service type at each facility level. The legend is ordered by the area of the rectangle.

Fig 3 (and S1 Fig) shows the annual (and monthly) frequency of each service type at each facility level. Across three levels, it is consistent that OPD, EPI, VCTTests and EstAdult were the most frequently delivered service types; while the least frequently delivered were U5Malnutr, NewAdult, PMTCT, MentalAll, DentalAll, TBNew and Csection. Consistent with our expectations of care at primary and secondary levels, some service types occurred mostly at level 1a (including AccidentsandEmerg, AntenatalTotal, Delivery, EPI, EstAdult, FamPlan, IPAdmission, MaleCirc, NewAdult, OPD, PMTCT, STI, U5Malnutr and VCTTests), whilst others occurred mostly at level 2 (Csection, DentalAll, InpatientDays, MentalAll and Peds) (see S1 Fig).

There was a clear temporal trend observed in some service types (see S1 Fig). Seasonal trends were noted in OPD visits, which tended to peak between January and May each year and the frequencies of Csection, EstAdult, FamPlan, PMTCT, STI, DentalAll and MentalAll services all increased annually.

### Changes in health service utilisation during the COVID-19 pandemic

**Fig 4:**
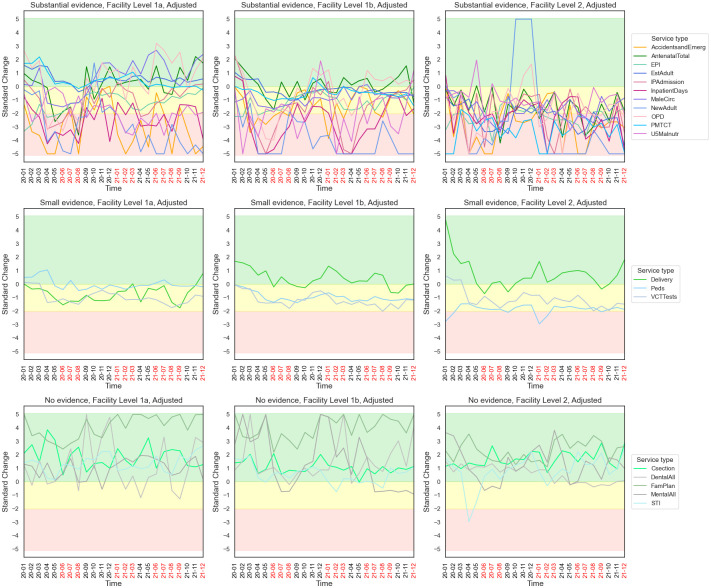
Standard monthly change in 2020–2021, per Test One. Red, yellow and green areas indicate substantial, small and no evidence of reductions, respectively, during the pandemic months. Service types were divided to three groups accordingly (also see Table 2). Standard change per service type per level is presented in S2 Fig.

A summary result of each test for each service type is provided in Table 2, wherein each service type was assigned “Substantial”, “Small” or “No” based on the evidence of reductions found in at least one facility level. Detailed results for each service type applying Test One are depicted in Fig 4 (and S2 Fig); and detailed results for each service type applying Test Two and Test Three are depicted in S3–S6 Figs. The results from all the tests were broadly consistent with one another.

**Table 2:** Evidence of reductions during pandemic months (April 2020-December 2021) per service type per test.

Service type	Test One	Test Two	Test Three
AccidentsandEmerg	Substantial	Substantial	Small
AntenatalTotal	Substantial	Substantial	Small
EPI	Substantial	Substantial	Substantial
EstAdult	Substantial	Substantial	Substantial
InpatientDays	Substantial	Substantial	Small
IPAdmission	Substantial	Substantial	Small
MaleCirc	Substantial	Substantial	Substantial
NewAdult	Substantial	Substantial	Substantial
OPD	Substantial	Substantial	Small
PMTCT	Substantial	Substantial	Substantial
U5Malnutr	Substantial	Substantial	Small
Delivery	Small	Small	Small
Peds	Small	Small	Substantial
VCTTests	Small	Small	Substantial
Csection	No	No	No
DentalAll	No	No	Substantial
FamPlan	No	No	No
MentalAll	No	No	Substantial
STI	No	No	No

For each service type within each test, “Substantial”, “Small” or “No” was assigned based on the consistent evidence of reductions found in at least one facility level among 1a, 1b and 2 during pandemic months of April 2020-December 2021. Some services had substantial evidence across all levels, such as OPD, whereas some others had substantial evidence at facility level 2, such as EstAdult and PMTCT. Refer to S2, S4, and S6 Figs in Supporting files for detailed reduction categories at each facility level.

Applying Test One and Test Two, we found substantial evidence of reductions for 11 service types including AccidentsandEmerg, AntenatalTotal, EPI, EstAdult, InpatientDays, IPAdmission, MaleCirc, NewAdult, OPD, PMTCT and U5Malnutr; and small evidence of reductions for 3 service types including Delivery, Peds and VCTTests. Other service types saw increasing frequencies at three facility levels instead during the pandemic period, including Csection, DentalAll, FamPlan, MentalAll and STI.

Applying Test Three, some differences were observed: among the 11 service types with substantial evidence of reductions per Test One, AccidentsandEmerg, AntenatalTotal, InpatientDays, IPAdmission, OPD and U5Malnutr had small evidence found; among the 3 service types with small evidence of reductions per Test One, Peds and VCTTests had substantial evidence found instead; among the 5 service types that showed no evidence of reductions per Test One, DentalAll and MentalAll had substantial evidence found. These differences are expected, as a higher per cent of facilities per Test Three does not necessarily indicate a greater extent of service frequency reduction per Test One (or Test Two), i.e., the reduced frequency at the majority of facilities might be cancelled out by the increased frequency at the minority of facilities.

The greatest reductions occurred in the period of April-August 2020, which coincided with the first wave of the COVID-19 pandemic in Malawi when the most stringent restrictions were in place. During this period, most service types (except U5Malnutr) for which there were substantial evidence for reductions across facility levels in Test One (and Test Two) saw these reductions repeated at all facility level 2 or multiple levels. For U5Malnutr, the substantial reductions were mostly at levels 1a and 1b in 2021. (See Fig 4 and S2 Fig).

From September 2020, rebounds were observed but with different degrees. OPD frequency recovered quickly and showed positive change by the end of 2020, whereas others including InpatientDay, IPAdmission, InpatientDays, AccidentsandEmerg, AntenatalTotal, EPI and MaleCirc showed slower rebounds. Despite these recoveries, however, the reductions of most service types persisted till the end of 2021. (See Fig 4 and S2 Fig).

We had only yearly data of TB case notifications (i.e., TBNew) in 2017–2021 and were not able to conduct monthly tests as for other service types. Nonetheless, we found certain reductions on TB case notifications using the limited data: in 2020, notified cases substantially reduced by approximately 10% and 13% at levels 1b and 2 respectively (more than 3 standard deviations below the mean values in 2017–2019); in 2021, notified cases substantially reduced by approximately 28% at level 2 (more than 5 standard deviations below the mean).

### Quantification in deficits of service utilisation during the COVID-19 pandemic

Table 3 presents the estimations of the cumulative health service deficits per service type at the national level during defined periods throughout 2020–2021, where values of each pandemic period were compared to average values of corresponding periods in 2015–2019.

**Table 3:** National-level absolute change per 1000 persons, percentage change and total deficit in 2020–2021, per Test One.

Service Type	Evidence of Reduction*	Apr—Aug 2020	Apr—Dec 2020	2021	Apr 2020—Dec 2021	Total Deficit†
AccidentsandEmerg	Substantial	-1.87 (-21.56%)	-2.47 (-15.5%)	-3.51 (-16.43%)	-5.98 (-16.03%)	-108,656
AntenatalTotal	Substantial	-2.68 (-9.15%)	-2.83 (-5.4%)	-0.19 (-0.27%)	-3.02 (-2.47%)	-54,860
EPI	Substantial	-70.85 (-25.89%)	-93.58 (-19.25%)	-105.83 (-16.37%)	-199.4 (-17.61%)	-3,622,599
EstAdult	Substantial	-5.97 (-6.53%)	-9.26 (-5.6%)	-7.23 (-3.31%)	-16.49 (-4.3%)	-299,520
InpatientDays	Substantial	-11 (-24.18%)	-16.08 (-20.25%)	-21.31 (-19.88%)	-37.39 (-20.03%)	-679,240
IPAdmission	Substantial	-6.04 (-16.39%)	-8.06 (-12.5%)	-8.23 (-9.46%)	-16.29 (-10.75%)	-295,961
MaleCirc	Substantial	-9.85 (-88.28%)	-9.75 (-60.49%)	5.5 (27.81%)	-4.25 (-11.84%)	-77,182
NewAdult	Substantial	-0.71 (-34.69%)	-0.79 (-22.19%)	-2.02 (-41.9%)	-2.82 (-33.52%)	-51,165
OPD	Substantial	-77.13 (-17.7%)	-62.14 (-8.35%)	33.59 (3.23%)	-28.55 (-1.6%)	-518,588
PMTCT	Substantial	-0.09 (-4.78%)	-0.07 (-2.16%)	-0.28 (-6.79%)	-0.35 (-4.79%)	-6,367
U5Malnutr	Substantial	-0.36 (-15.24%)	-0.96 (-23.37%)	-2.5 (-42.23%)	-3.46 (-34.5%)	-62,831
Delivery	Some	-0.17 (-1.5%)	-0.35 (-1.74%)	0.08 (0.31%)	-0.27 (-0.58%)	-4,889
Peds	Some	-5.15 (-40.37%)	-8.87 (-39.35%)	-13.1 (-42.77%)	-21.97 (-41.32%)	-399,115
VCTTests	Some	-29.21 (-31.84%)	-40.41 (-24.87%)	-63.41 (-29.2%)	-103.81 (-27.34%)	-1,885,982
Csection	No	0.18 (18.87%)	0.35 (20.15%)	0.5 (21.82%)	0.85 (21.09%)	15,523
DentalAll	No	1.82 (70.06%)	4.13 (82.99%)	0.49 (7.86%)	4.62 (41.17%)	83,996
FamPlan	No	52.07 (63.51%)	108.4 (73.15%)	204.81 (105.04%)	313.21 (91.27%)	5,690,196
MentalAll	No	0.24 (16.13%)	0.64 (24.79%)	1.49 (44%)	2.13 (35.71%)	38,710
STI	No	0.61 (8.79%)	2.46 (19.31%)	3.91 (23.18%)	6.37 (21.52%)	115,782
**Total deficits for appointments with substantial evidence of reduction**					-318 (-8.24%)	-5,776,970
**Total deficits for appointments with substantial or some evidence of reduction**					-444.05 (-10.23%)	-8,066,956

*This column is referred to Table 2.

^**†**^The total deficits are calculated for the period of April 2020-December 2021 and measure the total number of reduced services. The relative percentage change is the same to column April 2020-December 2021.

In the period of April 2020-December 2021, InpatientDays, NewAdult, U5Malnutr, Peds and VCTTests had cumulative reductions of more than 20%. Other service types were reduced, but more modestly: AccidentsandEmerg, EPI, IPAdmission and MaleCirc had reductions ranging from 10% to 20%, and AntenatalTotal, EstAdult, OPD, PMTCT and Delivery had reductions of less than 5%. The period April-August 2020 (with the highest Stringency Index and the first wave of infections) saw the greatest reductions, with later periods showing lesser deficits or rebounds in the frequency of services.

In changes per 1000 persons between April 2020-December 2021, the services most reduced were EPI and VCTTests, both of which were reduced by more than 100 counts per 1000 persons. Overall, the total number of services that were “lost” during the pandemic period is estimated to be 8 066 956 (10.23% reduction), which equated to 444 units per 1000 persons.

S7 Fig presents the national level changes and deficits applying Test Two, which are consistent with Table 3 and estimate a total deficit of 8 017 975 (10.52% reduction), which equated to 441 units per 1000 persons.

Finally, in Table 4, we summarised the national annual outpatient and inpatient service utilisation indicators in 2015–2021 in comparison with targets provided by the Malawi HHFA 2018/2019 report that refers to WHO guidelines [30]. In the pre-pandemic period, outpatient service utilisation was approximately 1 visit per person per year, which is 80% lower than the target; and hospital discharges averaged less than 4.5 per 100 persons per year, which is 55% lower than the target. For both service types, but especially for hospital discharges, the utilisation reduced in 2020 and 2021, making the actual frequencies even further from respective targets.

**Table 4:** OPD and Hospital discharges utilisation indicators 2015–2021.

Year	Outpatient visits per person per year		Hospital discharges per 100 persons per year	
	Adjusted	Unadjusted	Adjusted	Unadjusted
2015	1.04	0.99	4.33	4.23
2016	1.04	0.99	4.20	4.09
2017	1.05	0.95	4.25	3.90
2018	1.12	1.06	4.38	4.17
2019	1.09	1.01	3.95	3.78
2020	0.96	0.90	3.55	3.38
2021	0.99	0.93	3.53	3.36
**Target**	**5.00**		**10.00**	

## Discussion

We analysed monthly- and facility-based service utilisation data on various service types and described their utilisation at multiple facility levels within Malawi healthcare system in the years prior to the COVID-19 pandemic and during the pandemic. We found substantial evidence for reductions in many types of services–especially Accident & Emergency, EPI, inpatient stay, and HIV treatment. The reductions were greatest during the first wave of the pandemic when restrictions were most severe, but have persisted for some service types (e.g., U5Malnutri). More encouragingly, we found that some of the more frequent types of services (e.g., for delivery and maintaining persons on ART) were affected by a lesser extent. These signals emerge consistently when analysing the data under a variety of different assumptions to overcome incomplete data. We concluded that there has been an enormous net deficit of healthcare system utilisation in Malawi during the pandemic period, totalling around 8 million fewer services, leaving targets for healthcare utilisation even more distant than before the pandemic.

In fact, the true difference for services for health conditions other than the COVID-19 would be greater than these results indicated. This is because, the counts of generic inpatient and outpatient services, for which we detected reductions, will also include the treatment of patients for the COVID-19, which would have increased greatly during this time (as a reference, there were 88 086 confirmed COVID-19 cases in Malawi by December 2021 [22]).

The cause of the changes in healthcare system utilisation is not clear and could result from “supply side” (e.g., less clinic time, shifted health resources to pandemic-related activities) or “demand side” considerations (e.g. travel restrictions for persons that intended to seek health care, fears of contracting the COVID-19 that reduce health care seeking) [1–6]. Besides, the reductions in some service types like ART initiations and TB case notifications could be confounded with an actual decrease in incidence of these communicable diseases. According to reports by UNAIDS (The Joint United Nations Programme on HIV/AIDS) and Stop TB Partnership [31, 32], there were indeed decreasing trends for numbers of people developing TB and new HIV infections between 2010–2020 in Malawi. Also, we observed that some service types such as HIV treatment had consistently more reductions at secondary level than primary level from three tests (S2, S4 and S6 Figs); but the reasons behind are not known and could be that these services had been transferred to lower levels or that secondary level had to reserve more capacities for COVID-19 relevant services following the restriction policies. Such causes and confounding factors should be further investigated.

These findings complement other studies in Malawi that have found changes in single programmes like HIV, TB and neonatal care, or examined changes in a subset of health facilities [4–6, 16–19]. The overall picture that emerges does not match the worst-case scenarios considered at the outset of the pandemic, in which the most key services were reduced to very low levels [33–35]. Indeed, a degree of resilience–as defined by [36]–can be noted in the more modest reductions observed for the key service types.

Our study benefited from the electronic health information management systems in Malawi being reasonably complete for many service types, with a range of years of reliable data and readily accessible. However, our main concern in using these data was the biases induced through the incompleteness of data, rather than random sampling errors as the data should reflect a full account of all the services at all the facilities. First, data before 2015 were considered to have too low reporting rates (for multiple service types) to be used; even for data in 2015–2021, dental and mental service types were especially impacted by very low reporting rates. The low reporting rates could mean that findings for these services will be less certain and more at risk of being biased, detracting from the consistent picture that is built up of disruptions of many types of services that have high reporting rates. Second, some facilities that provide services did not provide any reports, as they are not expected to report to or not yet registered in the DHIS2 system (i.e., Type B and C missing data). Third, there were some service types like surgery, laboratory and TB follow-up visits [24, 28] not included in our analysis due to lack of data. Therefore, our approach to the analysis was to test the data in multiple ways under different assumptions of adjustment and then compare the results. This did provide reassurance that the signals detected were not entirely artefactual, but it is not possible to be definitive. Furthermore, we were aware that in the analysis, the total number of services delivered could be underestimated because there was no way to reflect the services that occur at facilities with no reports and we were unable to access data for those service types not included.

Finally, although we achieved the aims to analytically describe the magnitude and duration of changes in service utilisation across Malawi healthcare sector during the COVID-19 pandemic period of 2020–2021, further testing of the direct impacts of the disruptions and the actual mitigation policies on routine health services over the entire duration of the pandemic may be achieved by following the methods for the interrupted time series design and modelling analyses [6, 7, 18]. Furthermore, it would be interesting to relate changes in health service utilisation at primary and secondary levels to the changes of restrictions occurring in that period in a more disaggregated manner, in order to isolate the relative roles of different types of restrictions (e.g., clinics being too full to provide certain elements of care versus times when patients were otherwise disinclined to seek care).

## Conclusion

It is concerning to find reductions in healthcare service utilisation of this magnitude and duration as it would seem to indicate the possibility of substantial health losses could have occurred as a result. Some estimates have been developed already (as per [37], LMICs would see 1 157 000 additional child death and 56 700 additional maternal deaths over 6 months in the most severe scenario), which stand alongside earlier model projections that were formed before data on actual patterns of changes in health service utilisation were available [33, 34]. The empirical evidence provided in this study will provide a foundation upon which the health consequences of these changes in Malawi can be estimated, which we hope can be used to help guide steps taken to achieve a greater degree of health system resilience in the future.

## Supporting information

S1 Dataset The minimal datasets that derive the results in tables and plots. (XLSX)

S1 File The adjustment methods for the “Adjusted” dataset. (DOCX)

S1 Fig Monthly frequency per service type per facility level 2015–2019. (TIF)

S2 Fig Standard monthly change per service type per facility level in 2020–2021, per Test One. (TIF)

S3 Fig Standard monthly change in 2020–2021, per Test Two. (TIF)

S4 Fig Standard monthly change per service type per facility level in 2020–2021, per Test Two. (TIF)

S5 Fig Monthly per cent of facilities with reduced service frequency in 2020–2021, per Test Three. (TIF)

S6 Fig Monthly per cent of facilities with reduced frequency per service type per facility level in 2020–2021, per Test Three. (TIF)

S7 Fig National changes per 1000 persons (percentage change) and total deficits in 2020–2021, per Test Two. (TIF)
