# Supplementary material for: The changes in health service utilisation in Malawi during the COVID-19 pandemic
Source: PLoS One. 2024 Jan 17;19(1):e0290823. doi: 10.1371/journal.pone.0290823 (PMC10793884; doi:10.1371/journal.pone.0290823)
Supplement: S1 File — (DOCX) [file pone.0290823.s002.docx]

**The adjustment methods for the “Adjusted” dataset**

The records are the total number of services of a particular type $a$, at facility $f$, in month $m$ of year $y$: $u_{a,f,y,m}$; associated with reporting rates $r_{a,f,y,m}$ , which are binary variables, denote whether or not the record is reported.

**1. The formulas**

For each unreported record with $u_{a^{*},f^{*},y^{*},m^{*}}=0,$ $r_{a^{*},f^{*},y^{*},m^{*}}=0$ and $y^{*}$ in 2015-2019 (inclusive), the adjusted record ${u'}_{a^{*},f^{*},y^{*},m^{*}}$ is calculated as follows:

1. $We first apply M1,$

${u'}_{a^{*},f^{*},y^{*},m^{*}}= \frac{\sum_{y=2015}^{2019} (u_{a^{*},f^{*},y,m^{*}}\times r_{a^{*},f^{*},y,m^{*}})}{\sum_{y=2015}^{2019} r_{a^{*},f^{*},y,m^{*}}}$,

which fills-in unreported data through interpolating from the same facility and same month from other years with reported data.

1. $\mathrm{If} {u'}_{a^{*},f^{*},y^{*},m^{*}}=0, then apply M2$,

${u'}_{a^{*},f^{*},y^{*},m^{*}}= \frac{\sum_{m=1}^{12} (u_{a^{*},f^{*},y^{*},m}\times r_{a^{*},f^{*},y^{*},m})}{\sum_{m=1}^{12} r_{a^{*},f^{*},y^{*},m}}$,

which fills-in unreported data through interpolating from the same facility from other months of the same year with reported data.

1. $\mathrm{If} {u^{'}}_{a^{*},f^{*},y^{*},m^{*}}=0, then apply M3,$

${u'}_{a^{*},f^{*},y^{*},m^{*}}= \frac{\sum_{y=2015}^{2019} \sum_{m=1}^{12} (u_{a^{*},f^{*},y,m}\times r_{a^{*},f^{*},y,m})}{\sum_{y=2015}^{2019} \sum_{m=1}^{12} r_{a^{*},f^{*},y,m}}$,

which fills-in unreported data through interpolating from the same facility from all other months and years with reported data.

1. $\mathrm{If} {u^{'}}_{a^{*},f^{*},y^{*},m^{*}}=0, then apply M4,$

${u'}_{a^{*},f^{*},y^{*},m^{*}}= \frac{\sum_{\forall f \mathrm{with} p\left( f \right)=p^{*}} \sum_{y=2015}^{2019} \sum_{m=1}^{12} (u_{a^{*},f,y,m}\times r_{a^{*},f,y,m})}{\sum_{\forall f \mathrm{with} p\left( f \right)=p^{*}} \sum_{y=2015}^{2019} \sum_{m=1}^{12} r_{a^{*},f,y,m}}$,

which fills-in reported data through interpolating from other facilities of the same type $p\left( f \right)=p^{*}$ from all months and years with reported data.

(In each formula, if the denominator equals to zero, then $u^{'}=0$.)

For each aggregated frequency $u_{a,l,y,m}$ of service type $a$ at facility level $l$ in month $m$ of year $y$ between 2020 and 2021, we adjust it to ${u'}_{a,l,y,m}$ using formula M5 as follows:

1. $M5: {u'}_{a,l,y,m}= x_{a,l}\times u_{a,l,y,m}$,

where $x_{a,l}= \frac{{\sum_{y=2015}^{2019} \sum_{m=1}^{12} {u'}_{a,l,y,m}}/{\sum_{y=2015}^{2019} \sum_{m=1}^{12} 1}}{{\sum_{y=2015}^{2019} \sum_{m=1}^{12} u_{a,l,y,m}}/{\sum_{y=2015}^{2019} \sum_{m=1}^{12} 1}}$ is the defined adjustment factor per service type per facility level.

**2. The implementation to data**

The table below shows the detailed implementation of the adjustment methods to each service type, considering both reporting-rates related data completeness and comparability to published data if available. Generally, M1-M4 were subsequently applied to services types that have high reporting rates (>79%); EPI data were not adjusted as the unadjusted data were comparable with WHO/UNICEF Joint Reporting Form on Immunization[1]; Male circumcision data only implemented M2 so that the adjusted data were comparable to Global AIDS monitoring 2021[2, 3]; TBNew data were not adjusted as the unadjusted data were comparable to National Tuberculosis Control Programme Annual Report[4]; Dental and mental data were not adjusted because of very low reporting rates; Service data from HIV Dept were not adjusted as no reporting rates are provided.

1. World Health Organisation. WHO/UNICEF Joint Reporting Form on Immunization (JRF). Available from: <https://www.who.int/teams/immunization-vaccines-and-biologicals/immunization-analysis-and-insights/global-monitoring/who-unicef-joint-reporting-process>.

2. UNAIDS and WHO. Uneven progress on the voluntary medical male circumcision. 2022.

3. UNAIDS. Global AIDS Monitoring 2021. Available from: <https://www.unaids.org/en/global-aids-monitoring>.

4. Government of Malawi. Ministry of Health National Tuberculosis Control Programme Annual Report. 2019.
