# Supplementary figures and images for: The changes in health service utilisation in Malawi during the COVID-19 pandemic
Source: PLoS One. 2024 Jan 17;19(1):e0290823. doi: 10.1371/journal.pone.0290823 (PMC10793884; doi:10.1371/journal.pone.0290823)

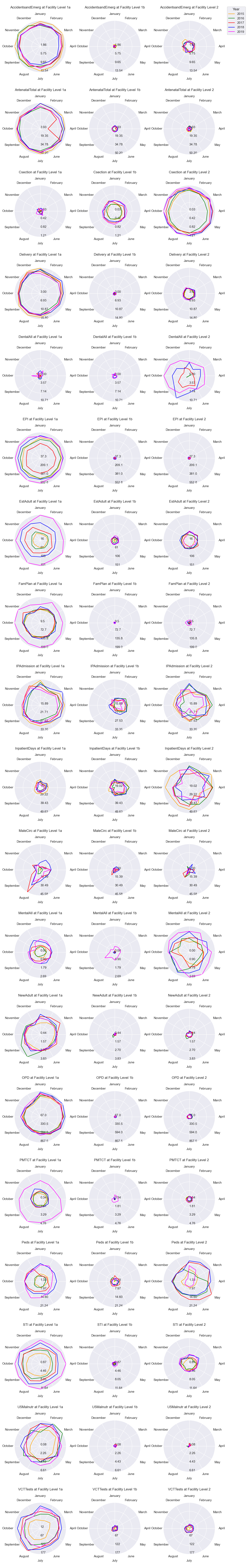

Supplement: S1 Fig — (TIF) [file pone.0290823.s003.tif]

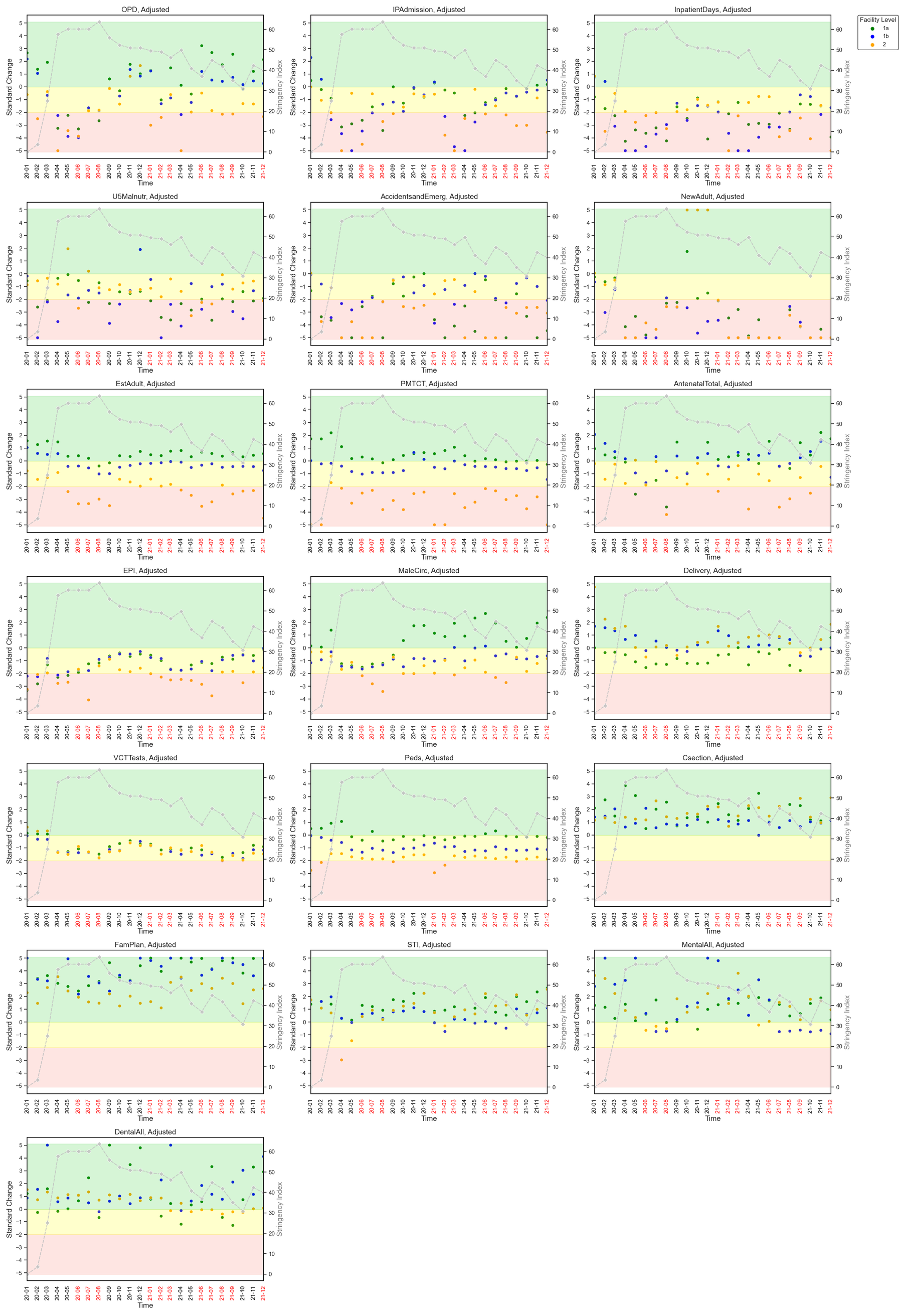

Supplement: S2 Fig — (TIF) [file pone.0290823.s004.tif]

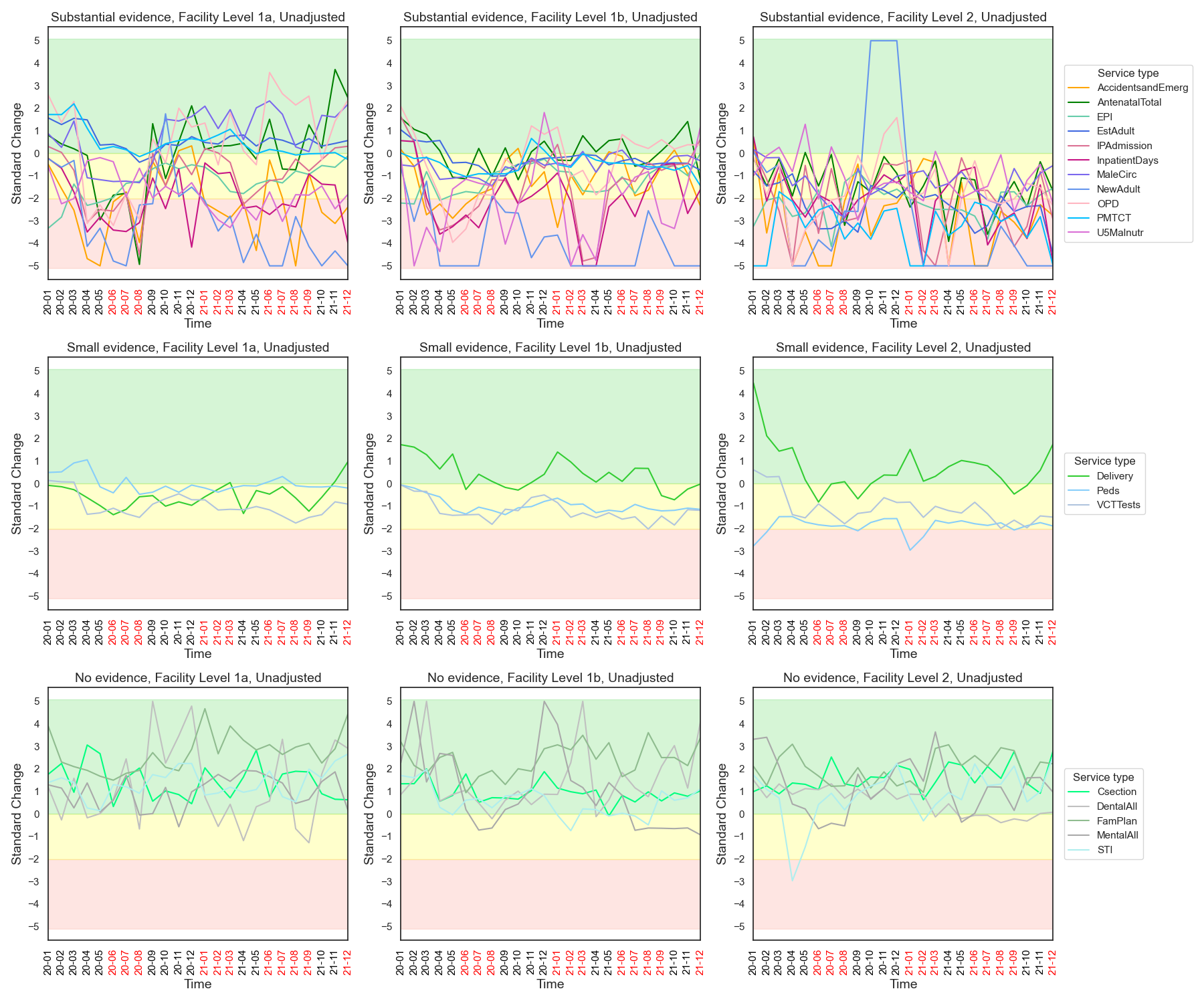

Supplement: S3 Fig — (TIF) [file pone.0290823.s005.tif]

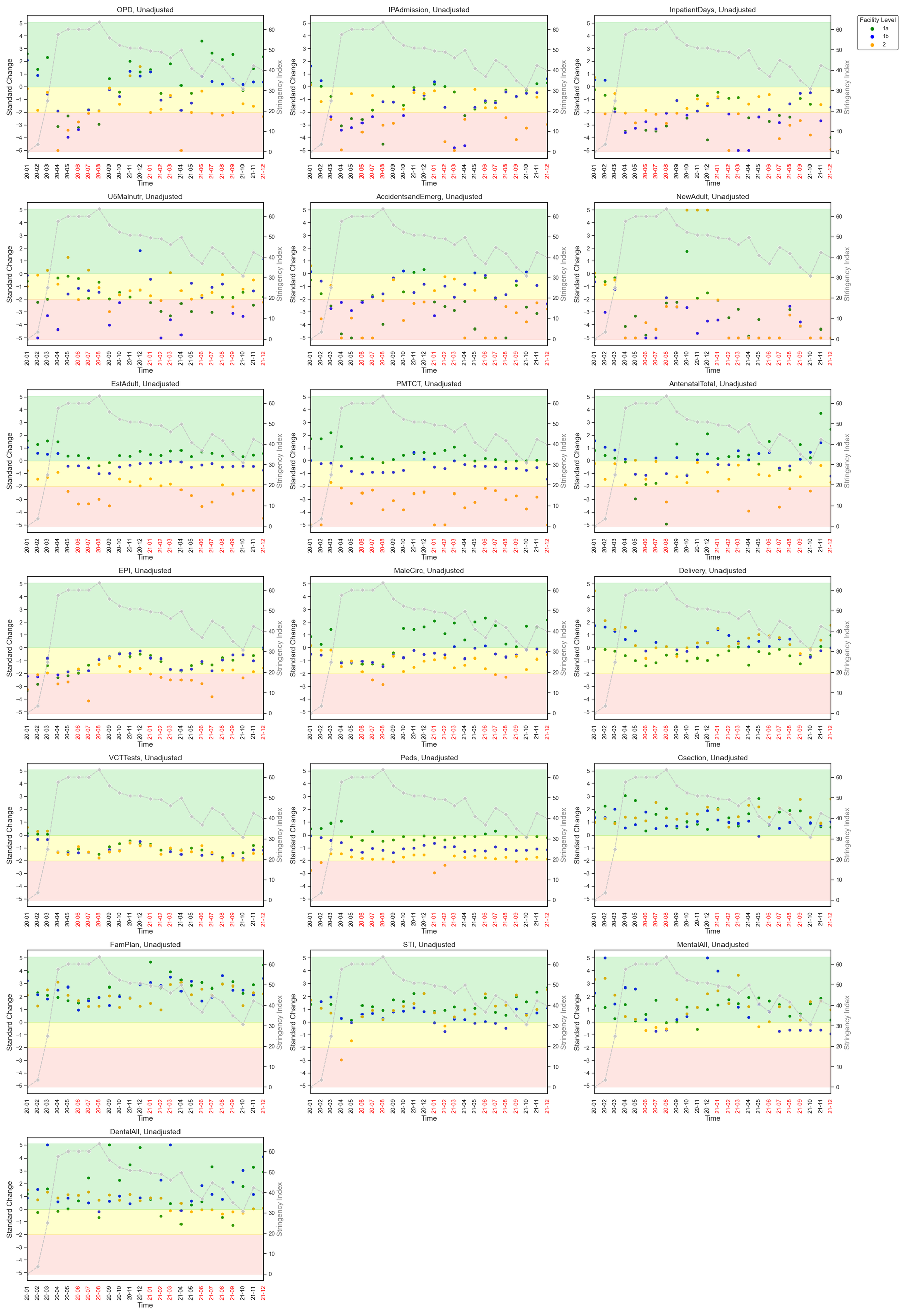

Supplement: S4 Fig — (TIF) [file pone.0290823.s006.tif]

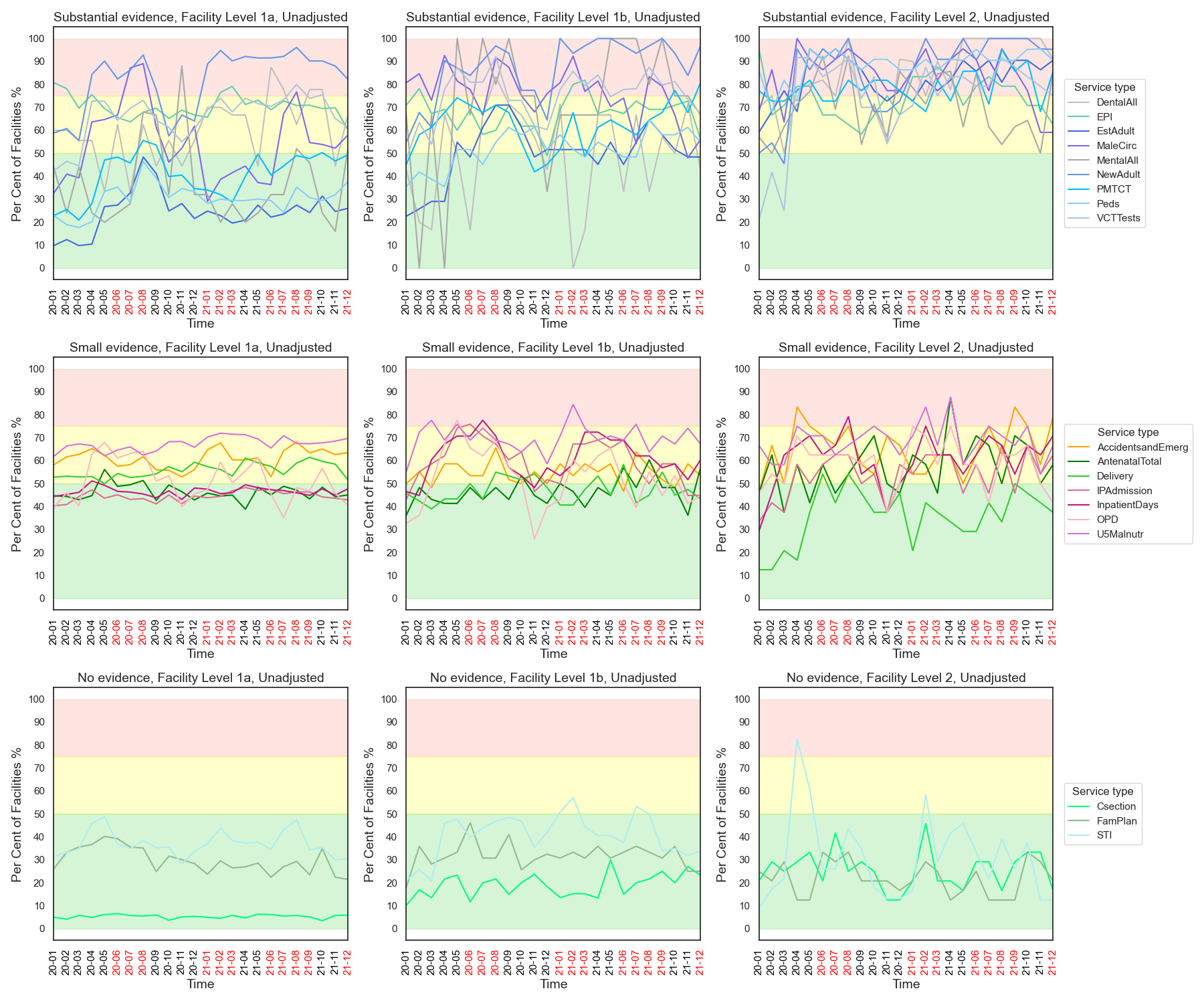

Supplement: S5 Fig — (TIF) [file pone.0290823.s007.tif]

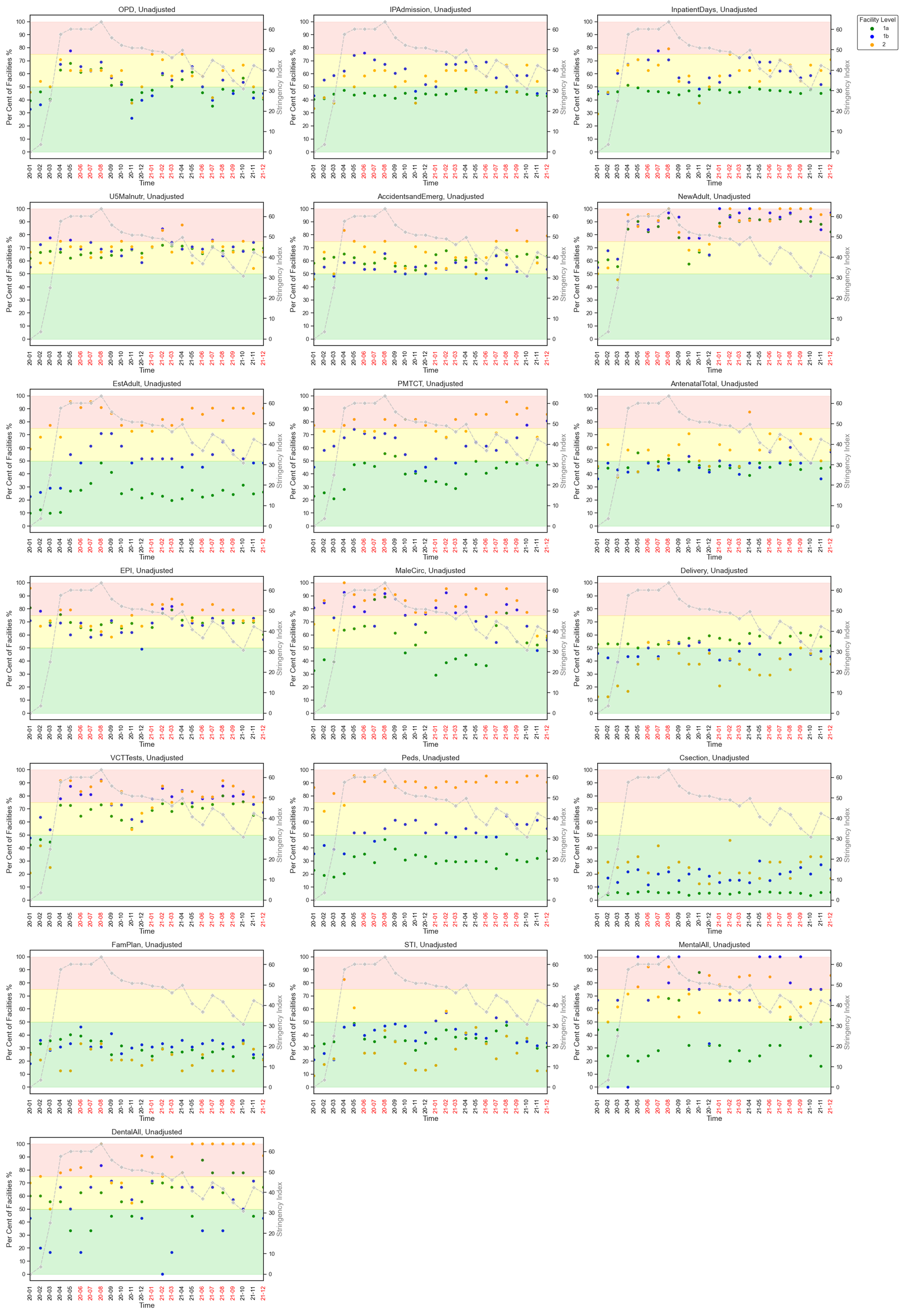

Supplement: S6 Fig — (TIF) [file pone.0290823.s008.tif]

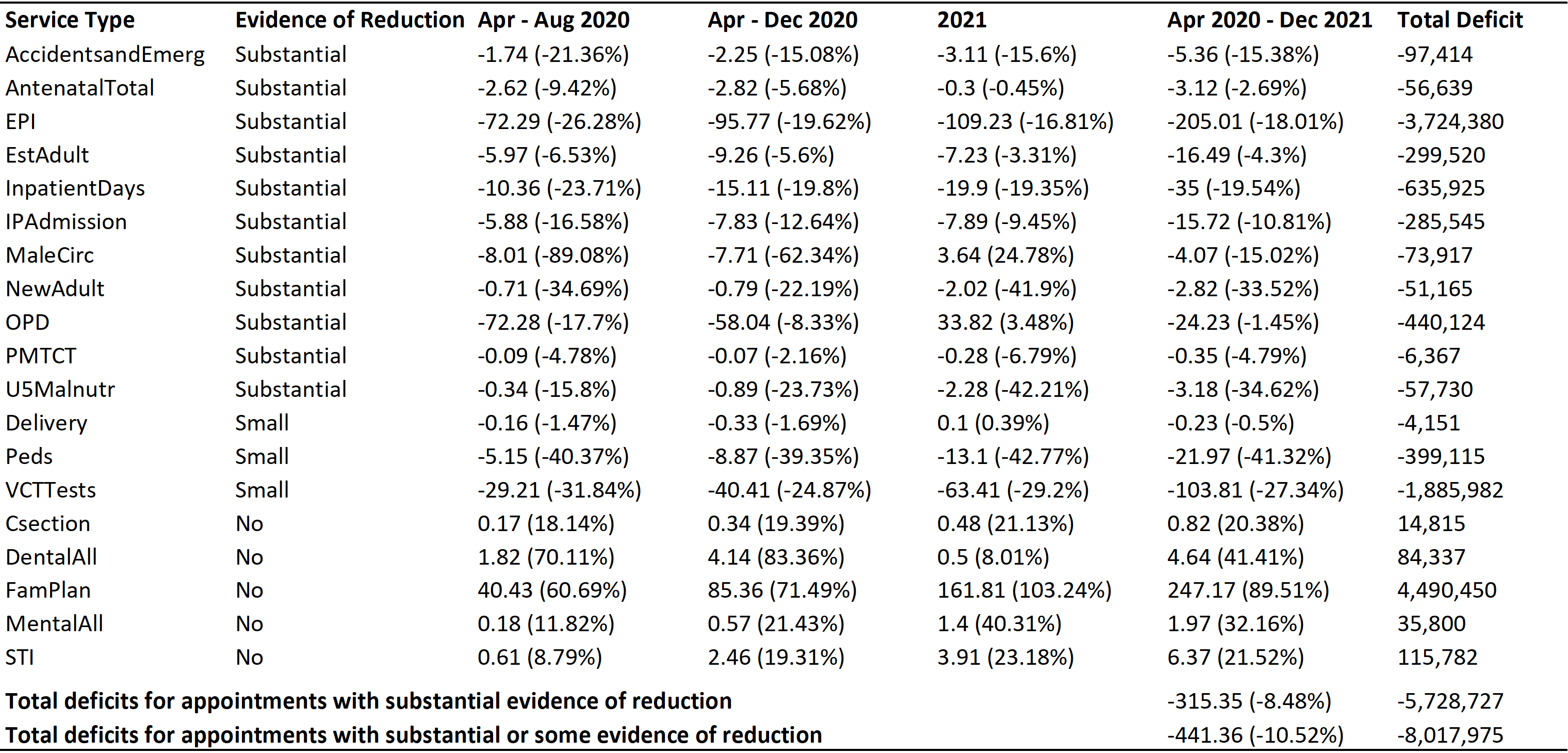

Supplement: S7 Fig — (TIF) [file pone.0290823.s009.tif]
